# Supplementary material for: CD301b lectin expression in the breast tumor microenvironment augments tumor growth
Source: Res Sq. 2025 Dec 3:rs.3.rs-8148605. Preprint. [Version 1] doi: 10.21203/rs.3.rs-8148605/v1 (PMC12687833; doi:10.21203/rs.3.rs-8148605/v1)
Supplement: Supplement 1 [file NIHPPRS8148605V1-supplement-1.pdf]

Supplementary Figures:

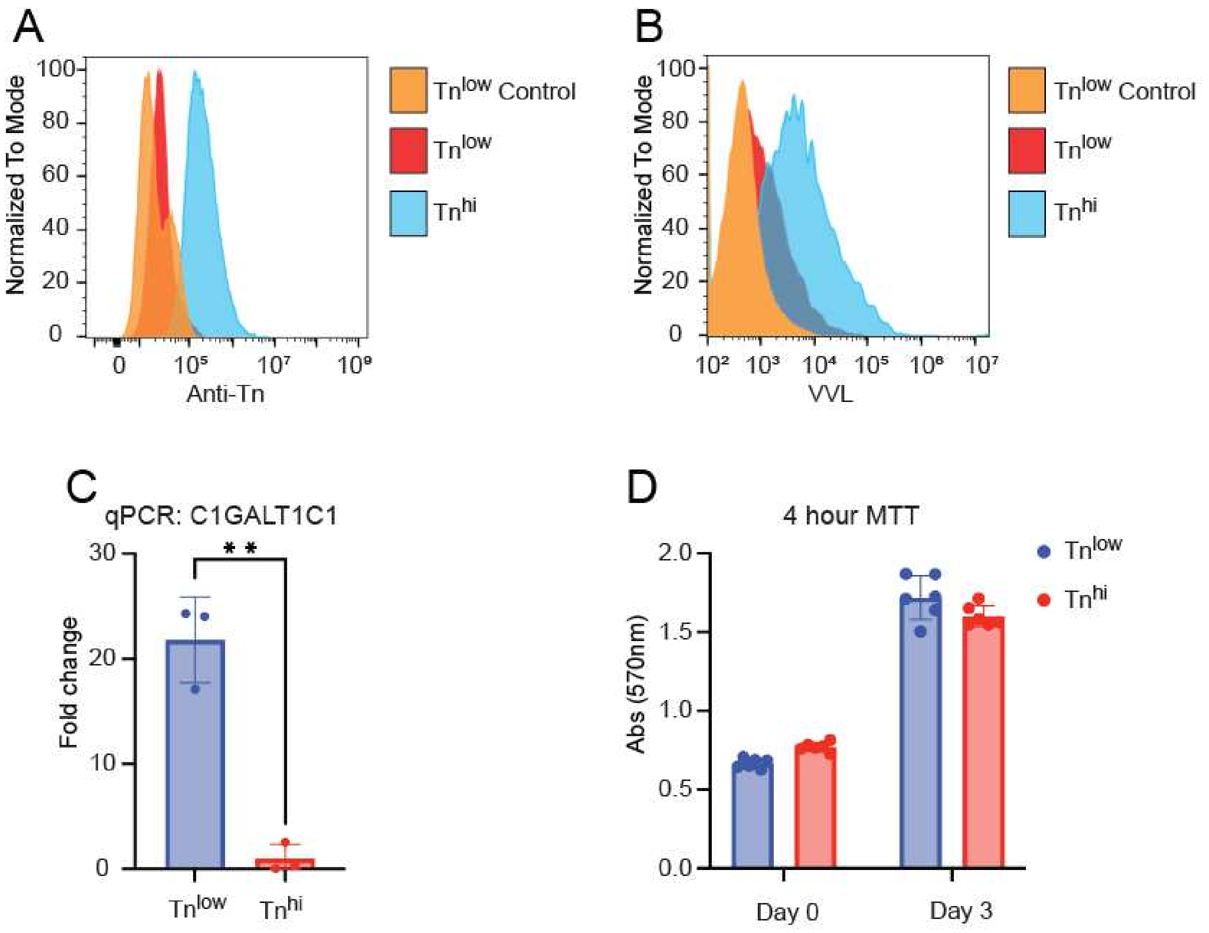

Supplementary Fig. 1. Generation of AT3 *Cosmc* KO (Tn^{hi}) murine breast cancer cells. **A. and B.** CRISPR-Cas9 KO of Cosmc chaperone increases Tn cell surface expression in AT3 murine breast cancer cells. AT3 (Tn^{low}) and AT3 *Cosmc* KO (Tn^{hi}) cells were stained with **A.** mouse anti-Tn (reBaGs6 IgM) primary and anti-IgM secondary antibodies, with secondary antibody-only as a control, and **B.** biotinylated VVL followed by fluorescently-conjugated streptavidin, with fluorescently-conjugated streptavidin-only as a control. **C.** RNA was isolated from AT3 (Tn^{low}) and AT3 *Cosmc* KO cells (Tn^{hi}). qPCR was performed in triplicate wells using validated *Cosmc* qPCR primers and using b-actin as a control for each sample. Fold changes were normalized against the average of AT3 *Cosmc* KO cells (Tn^{hi}). **D.** AT3 (Tn^{low}) and AT3 *Cosmc* KO cells (Tn^{hi}) were cultured in 96 well plates (n=5) at 1E4 cells/200mL for 3 days at 37oC. MTT proliferation assay was performed to determine cell growth.

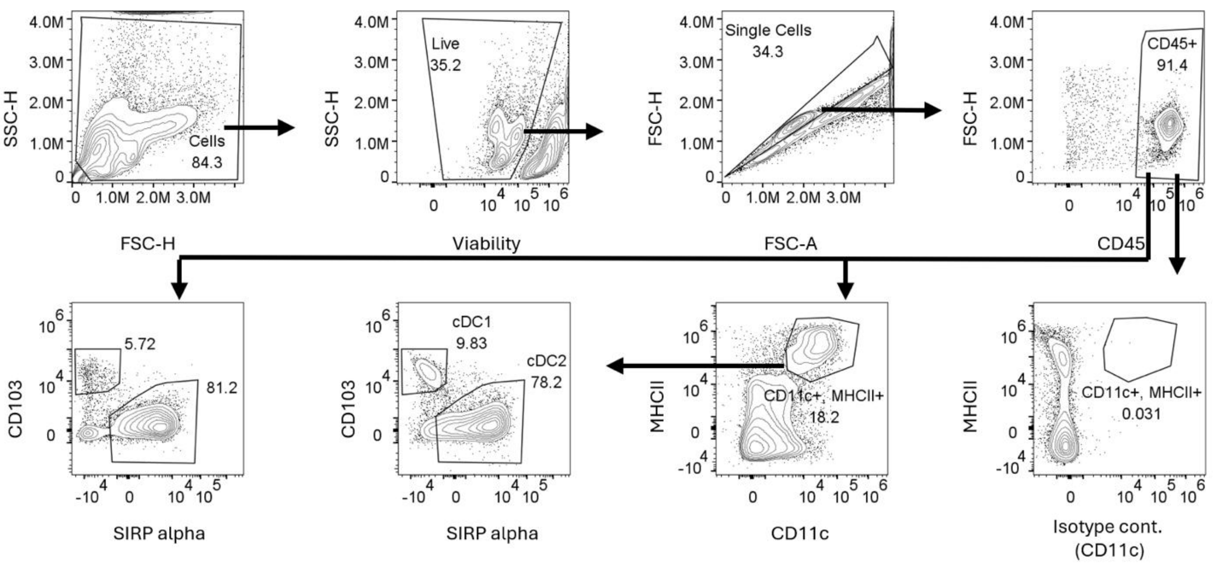

Supplementary Fig. 2. Flow cytometry gating strategy for identifying dendritic cells and their subsets. DCs were identified by expressions of MHCII and CD11c (bottom, middle, right) among $CD45^{+}$ cells (top, right). Isotype control of CD11c antibody was used to locate $CD11c^{+}$ cells (bottom, right). cDC1s and cDC2s were identified by expressions of CD103 and SIRP-alpha (bottom, middle, left). Total $CD45^{+}$ cells were used to locate $CD103^{-/+}$, and SIRP-$alpha^{-/+}$ cells (bottom, left).

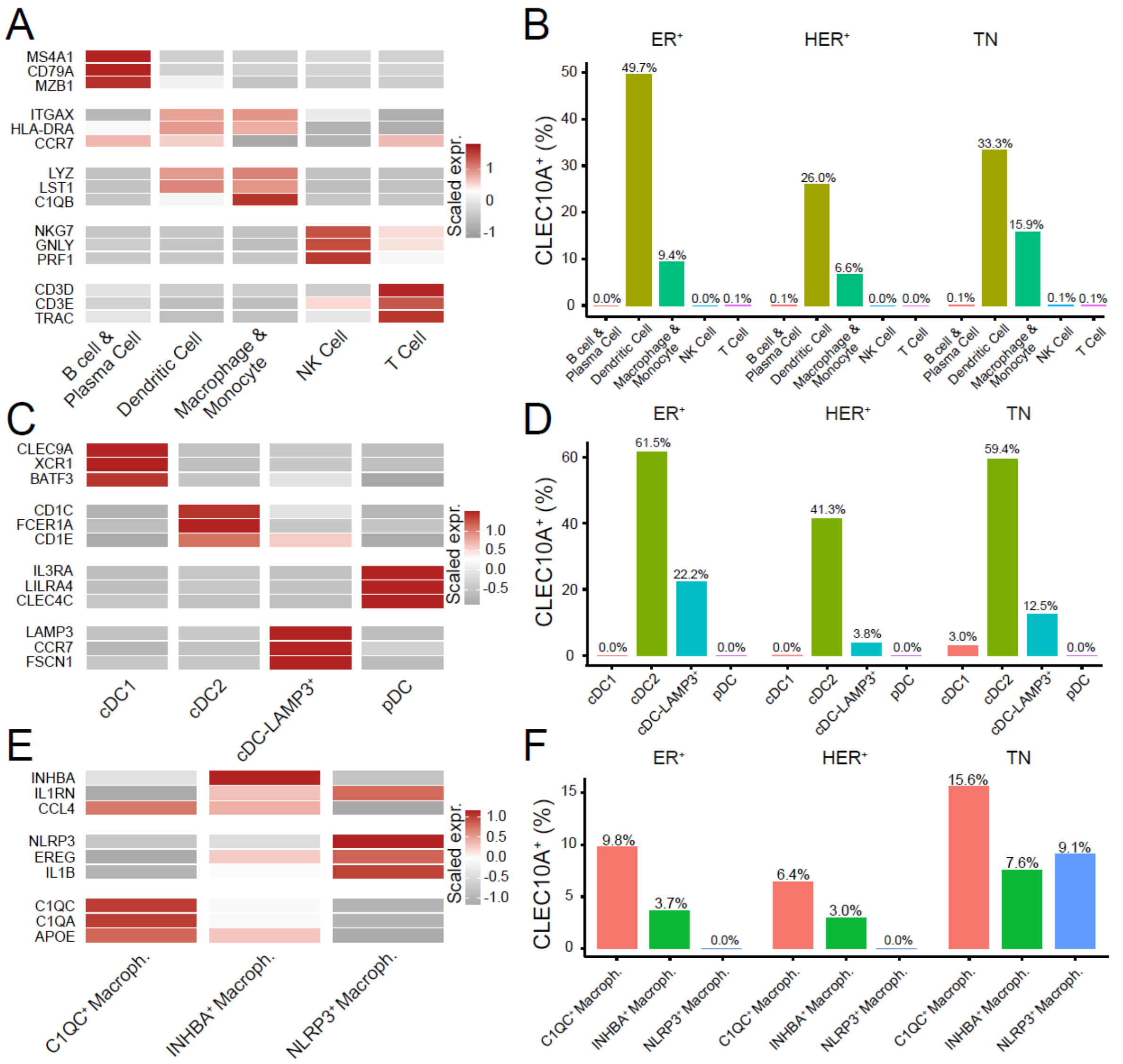

Supplementary Fig. 3. A. Heatmap showing canonical markers used to define major immune cell lineages (B/Plasma cell, Dendritic cell, Macrophage/Monocyte, NK cell, and T cell). **B.** Bar plots showing the frequency of $CLEC10A^+$ cells across major immune lineages in ER^+, $HER2^+$, and triple-negative (TN) breast cancers. **C.** Heatmap displaying canonical dendritic cell subset markers used to define cDC1, cDC2, cDC-$LAMP3^+$, and pDC populations. **D.** Corresponding bar plots showing $CLEC10A^+$ proportions within each DC subset across ER^+, $HER2^+$, and TN tumors. **E.** Heatmap showing markers used to define macrophage polarization and functional states, including $C1QC^+$ macrophages, $INHBA^+$ macrophages, and $NLRP3^+$ macrophages. **F.** Bar plots showing the proportion of $CLEC10A^+$ cells within each macrophage subtype across ER^+, $HER2^+$, and TN breast cancers.

Heatmaps display scaled average expression per cell group. Bar plots represent the percentage of $CLEC10A^+$ cells within each indicated population.
